# Metabolic surgery improves insulin resistance through the reduction of gut-secreted heat shock proteins

**DOI:** 10.1038/s42003-018-0069-8

**Published:** 2018-06-13

**Authors:** Giulia Angelini, Serenella Salinari, Alessandro Bertuzzi, Amerigo Iaconelli, Geltrude Mingrone

**Affiliations:** 1 Department of Internal Medicine, Catholic University, Largo A. Gemelli 8, 00168 Rome, Italy; 2 Department of Computer, Control, and Management Engineering “Antonio Ruberti”, University of Rome “Sapienza”, Via Ariosto 25, 00185 Rome, Italy; 3 CNR-Institute of Systems Analysis and Computer Science (IASI), Via dei Taurini 19, 00185 Rome, Italy; 4 Diabetes and Nutritional Sciences, Hodgkin Building, Guy’s Campus, King’s College London, London, UK

## Abstract

Metabolic surgery improves insulin resistance and is associated with the remission of type 2 diabetes, but the mechanisms involved remain unknown. We find that human jejunal mucosa secretes heat shock proteins (HSPs) in vitro, in particular HSP70 and GRP78. Circulating levels of HSP70 are higher in people resistant to insulin, compared to the healthy and normalize after duodenal–jejunal bypass. Insulin sensitivity negatively correlates with the plasma level of HSP70, while body mass index does not. A high-energy diet increases the circulating levels of HSP70 and insulin resistance. HSP70 stimulates the accumulation of lipid droplets and inhibits Ser473 phosphorylation of Akt and glucose uptake in immortalized liver cells and peripheral blood cells. Serum depleted of HSPs, as well as the serum from the insulin-resistant people subjected to a duodenal–jejunal bypass, reverse these features, identifying gut-secreted HSPs as possible causes of insulin resistance. Duodenal–jejunal bypass might reduce the secretion of HSPs either by shortening the food transit or by decreasing the fat stimulation of endocrine cells.

## Abstract (web summary)

Giulia Angelini et al. demonstrate that calorie-dense diets overstimulate the secretion of heat shock proteins from the small intestine, increasing insulin resistance. This study suggests that bariatric surgery improves insulin resistance by reducing the circulatory level of gut-secreted heat shock proteins.

## Introduction

Insulin resistance (IR) is the main disturbance observed in type 2 diabetes (T2D) that develops long before insulin secretion failure^1^. Non-alcoholic fatty liver disease (NAFLD) represents the hepatic manifestation of insulin resistance^2^ and its prevalence is particularly elevated in obese subjects^3^. In subjects with NAFLD, genes involved in lipid metabolism, especially perilipins (PLIN) gene family, are up-regulated^4^. Perilipin-2 (Plin2) is an isoform expressed in the liver of perilipin proteins surrounding lipid droplets. Plin2 controls lipid storage, hydrolysis and metabolic functions and its degradation is essential for lipolysis of lipid droplets^5^. In fact, reduced Plin2 expression by antisense oligonucleotides in the liver of *ob/ob* mice or of mice under a high-fat diet prevents hepatic lipid accumulation^6^ and Plin2-null mice do not develop hepatosteatosis when fed with a high-fat diet^7^.

Given the central role played by IR in both T2D and NAFLD, the discovery of the mechanisms through which IR develops would be of primary importance. Recently, metabolic surgery showed that the small intestine is strictly involved in IR. In fact, its bypass is associated with a prompt reversal of IR^8^ as well as of T2D^9–13^ and NAFLD^14^. These findings suggest that the small intestine of obese subjects can respond to nutrient overload by secreting hormones that induce insulin resistance. Recently, we have demonstrated that oral insulin sensitivity was considerably lower compared to intravenous glucose administration and that the bypass of the small intestine reverses these effects in obese subjects^15^. The duodenum and jejunum, indeed, secrete proteins that are able to determine IR either in vitro or in vivo^16^. That IR is an acquired condition depending on circulating factors is supported by IR complete reversion in adipocytes from T2D and IR subjects when studied in vitro after a 24-h washout^17^. Also muscles from insulin-resistant T2D rodents become completely insulin responsive in vitro^18^, suggesting the presence of humoral factor/s inhibiting glucose uptake. Lack of stimulation of the small intestine by nutrients, such as during intermittent fasting, improves insulin-mediated whole-body glucose uptake and lipolysis^19^. In addition, fasting in diabetic mice ameliorates insulin secretion^20^. A link between the small intestine and IR might be the secretion of extracellular stress proteins, such as heat shock proteins (HSPs) including glucose-regulated protein (GRP), which are emerging as important mediators of intercellular signaling in response to nutrients. Serum HSP70 levels are higher in diabetic patients than in normal subjects and correlate with diabetes duration^21^. Circulating HSP70 levels in gestational diabetes well correlate with the levels of glycated hemoglobin (HbA1c)^22^.

Furthermore, extracellular HSP70 induces the activation of several pro-inflammatory pathways possibly through the membrane Toll-like receptors (TLRs)^23^. TLR2/4-dependent activation of stress-activated c-Jun N-terminal kinase (JNK) promotes phosphorylation of the insulin receptor substrate-1 (IRS-1) at Ser307 in rodents leading to inhibition of Akt activation^24^ and, consequently, to a reduced insulin-mediated glucose uptake, at least in animal models.

We hypothesized that nutrient overload in obese subjects can cause gut cell stress that stimulates HSP secretion into the portal vein system leading to hepatic insulin resistance and NAFLD and, in the long term, to whole body IR. We also hypothesized that metabolic surgery with duodenal–jejunal bypass can reverse these features.

In this study, we have investigated the effects of metabolic surgery on circulating levels of HSPs and on insulin sensitivity. We show that in vitro stimulation with HSP70 and GRP78, as well as serum from obese IR subjects with NAFLD, impairs insulin-mediated glucose uptake and insulin signaling and stimulates fat accumulation in HepG2, circulating peripheral blood mononuclear cells (PBMCs) and monocyte subpopulations. We chose to study PBMCs because they are equipped with a set of TLRs^25^ and scavenger receptors^26^ removing and accumulating lipids in lipid droplets surrounded by Plin2^27^.

Furthermore, we proved HSP involvement in IR and NAFLD onset by HSP70 and GRP78 serum immune depletion. Our results point to gut-secreted HSPs as possible mediators of insulin resistance and suggest that their reduction in the circulatory stream might account for the beneficial effects of metabolic surgery.

## Results

### HSP secretion in the gut conditioned medium

**Fig. 1:**
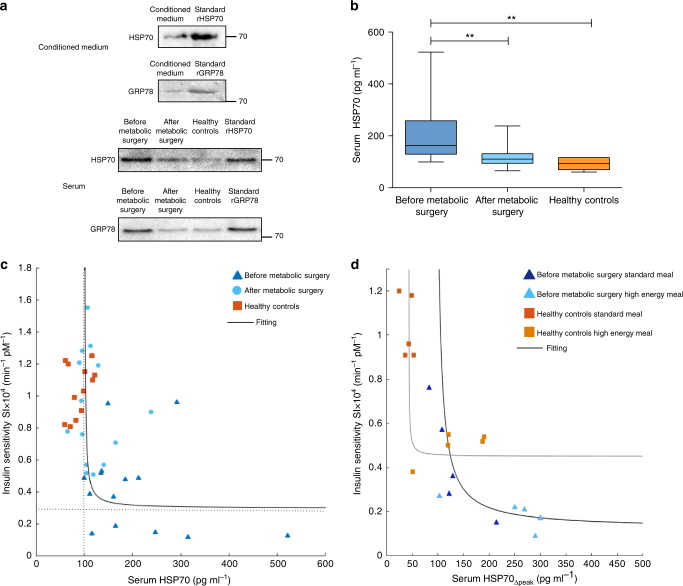
HSPs in conditioned medium and in plasma, and correlation with insulin sensitivity. **P<0.009. Data are mean ± s.e.m. or are expressed as median plus minimum and maximum values for whisker plots. **a** A representative western blot from one obese IR, NAFLD subject showing HSP70 and GRP78 in the conditioned medium of jejunal biopsies (top). HSP70 and GRP78 in the serum of a representative obese IR, NAFLD subject before and after metabolic surgery, and in the serum of one healthy control (bottom). Number of experiments *n* = 5. Original blot images are reported in the Supplementary Fig. 6. **b** HSP70 serum concentration in 14 obese IR, NAFLD subjects before and after metabolic surgery (bilio-pancreatic diversion (BPD)) and in 12 healthy controls (before metabolic surgery vs. after metabolic surgery: *P* = 0.007; before metabolic surgery vs. healthy controls: *P* = 0.008). **c** Insulin sensitivity (SI) was computed from OGTT data of obese IR subjects with NAFLD before and 6 months after surgery (*n* = 14) and of healthy controls (*n* = 12) by the oral glucose minimal model analysis and is plotted vs. fasting HSP70 serum concentration of the same subjects. Fitting line obtained by nonlinear regression: (HSP70 − HSP70_∞_) × (SI−SI_∞_) = *k*, with HSP70_∞_ = 100.14 pg mL^−1^, SI_∞_ = 0.30 × 10^4^ min^−1^ pM^−1^, *k* = 2.51 pg mL^−1^ min^−1^ pM^−1^, α < 0.05. The vertex of the hyperbole is at HSP70 = 127 pg mL^−1^ and SI = 0.38 × 10^4^ min^−1^ pM^−1^. **d** Insulin sensitivity (SI) is higher during a standard than during a high-energy breakfast (Table 1) in a subset of 5 obese IR, NAFLD subjects (*P* = 0.043) and in 5 healthy controls (*P* = 0.008). Insulin sensitivity (SI) is plotted vs. the incremental peak concentration of serum HSP70 (value at 60 min minus initial value, HSP70_Δpeak_) measured during the meal test. SIis negatively correlated with the HSP70 incremental peak level. Parameters of fitting lines: Obese: HSP70_Δpeak_,_∞_ = 93.9 pg mL^−1^, SI_∞_ = 0.12 × 10^4^ min^−1^ pM^−1^, *k* = 10.04 pg mL^−1^ min^−1^ pM^−1^, α < 0.05. Controls: HSP70_Δpeak_,_∞_ = 42.54 pg mL^−1^, SI_∞ _= 0.45 × 10^4^ min^−1^ pM^−1^, *k* = 0.50 pg mL^−1^ min^−1^ pM^−1^, α < 0.05

HSP70 and GRP78 were detected in the conditioned medium of jejunal biopsies of obese IR, NAFLD subjects. HSP70 and GRP78 were also found in sera from obese IR, NAFLD subjects before and after metabolic surgery and in sera from healthy controls (Fig. 1a).

### Reduction of circulating levels of HSP70 after metabolic surgery

To determine if metabolic surgery influenced HSP70 circulating levels, HSP70 was measured in the sera of obese IR, NAFLD subjects before and after metabolic surgery as well as in the sera of healthy controls. As shown in Fig. 1b, metabolic surgery significantly reduced the fasting HSP70 serum levels (from 203.2 ± 30.1 to 119.1 ± 11.1 pg mL^−1^, *P* = 0.007) that became comparable to the concentrations of healthy controls (119.1 ± 11.1 vs. 93.0 ± 10.7 pg mL^−1^, *P* = NS).

HSP70 circulating levels significantly and positively correlated with HOMA-IR (before metabolic surgery: *R* = 0.39, *P* = 0.029; after metabolic surgery: *R* = 0.66, *P* = 0.0004; healthy controls: *R* = 0.66, *P* = 0.0004) (Supplementary Fig. 1).

A negative hyperbolic correlation of HSP70 with whole body insulin sensitivity (SI)—measured by the oral glucose minimal model after an oral glucose tolerance test (OGTT)—was observed (Fig. 1c). Whole body insulin sensitivity was normalized after metabolic surgery, becoming similar to that of healthy controls (Table 1; Fig. 1c).

**Table 1:** Clinical characteristics, insulin sensitivity and secretion of the study population (mean ± s.d.)

	Before metabolic surgery	*P* value before/after	After metabolic surgery	*P* value before surgery and controls	Healthy controls	*P* value after surgery and controls
Number of patients	14		14		12	
Age (years)	41.4 ± 2.2	NS	41.4 ± 2.2	NS	40.1 ± 3.0	NS
Weight (kg)	142.4 ± 6.6	0.0002	108.5 ± 7.5	<0.0001	64.8 ± 3.8	0.0007
BMI (kg m^−2^)	49.1 ± 6.6	0.0002	36.4 ± 2.0	<0.0001	21.7 ± 0.8	<0.0001
Plasma glucose (mg dL^−1^)	101.0 ± 3.7	0.02	92.2 ± 9.8	0.002	76.4 ± 2.2	NS
Plasma insulin (mU L^−1^)	16.1 ± 2.1	0.02	8.5 ± 1.3	0.03	8.5 ± 0.68	NS
HbA1c (mmol mol^−1^)	41.6 ± 0.8	0.01	38.2 ± 0.6	<0.0001	25.5 ± 1.1	0.002
HOMA-IR	3.9 ± 0.5	0.01	1.6 ± 0.2	0.006	1.6 ± 0.1	NS
HDL-cholesterol (mg dL^−1^)	45.9 ± 2.1	0.005	34.9 ± 2.3	0.0008	33.4 ± 1.8	NS
LDL-cholesterol^a^ (mg dL^−1^)	106.8 ± 4.3	<0.0001	65.9 ± 3.4	<0.0001	43.6 ± 4.2	0.002
Total cholesterol (mg dL^−1^)	172.4 ± 5.3	0.002	146.4 ± 8.9	<0.0001	138.9 ± 5.7	NS
Triglycerides (mg dL^−1^)	129.8 ± 13.0	0.003	100.4 ± 14.2	0.01	86.2 ± 4.5	NS
ALT (IU L^−1^)	35.6 ± 4.7	0.001	25.1 ± 3.3	0.05	17.7 ± 0.6	NS
Glucose effectiveness (SG × 10^2^ min^−1^)	3.43 ± 0.31	NS	3.21 ± 0.35	NS	3.74 ± 0.21	NS
Insulin sensitivity (SI × 10^4^ min^−1^ pM^−1^)	0.42 ± 0.07	0.00012	0.96 ± 0.09	<0.0001	1.04 ± 0.23	NS
Total β-cell glucose sensitivity (Φ × 10^9 ^min^−1^)	67.69 ± 4.95	0.00012	45.57 ± 4.11	<0.0001	40.7 ± 5.8	NS

	Meal Test					
	Obese IR, NAFDL subjects			Controls		
	Standard		High-energy breakfast	Standard		High-energy breakfast
Number of subjects	5		5	5		5
Glucose effectiveness (SG × 10^2^ min^−1^)	3.70 ± 0.51	NS	4.40 ± 0.37	3.44 ± 0.20	NS	3.57 ± 0.65
Insulin sensitivity (SI × 10^4^ min^−1^ pM^−1^)	0.42 ± 0.11	0.043	0.19 ± 0.03	1.03 ± 0.06	0.008	0.50 ± 0.03

^a^LDL-cholesterol (mg dL^−1^) = total cholesterol (mg dL^−1^) − HDL-cholesterol (mg dL^−1^) – triglycerides (mg dL^−1^)/5^41^

In a multiple regression analysis including all subjects studied (*R* = 0.66, *P* = 0.010) with HSP70 as dependent variable and body mass index (BMI), SI and Homeostatic Model Assessment of Insulin Resistance (HOMA-IR) as independent ones, SI and HOMA-IR independently predicted the plasma levels of HSP70 with *P* = 0.0045 and *P* = 0.0056 respectively, while BMI was not significant (*P* = 0.065).

### HSP70 levels increase after high-energy breakfast

Supplementary Figure 2A shows that the high-energy breakfast significantly raised HSP70 serum levels after 60 min as compared to the standard diet in a subset of 5 obese IR, NAFLD subjects and 5 healthy controls (before surgery: 481.6 ± 22.5 vs. 363.4 ± 25.9 pg mL^−1^, *P* = 0.027; healthy controls: 212.0 ± 19.0 vs. 119.1 ± 5.2 pg mL^−1^, *P* = 0.019). The high-energy breakfast reduced whole body insulin sensitivity in obese IR, NAFLD subjects (*P* = 0.043) and in 5 healthy controls (*P* = 0.008) (Table 1). Serum HSP70 incremental peak inversely correlated with SI according to hyperbolic functions in obese IR and control subjects (Fig. 1d). HSP70 circulating levels significantly and positively correlated with plasma glucose, insulin, C-peptide, triglycerides and Plin2 protein expression in PBMCs as shown by linear regression analyses (Supplementary Figure 2B-F).

### Metabolic surgery reduces HSP70 and Plin2 mRNA expression in PBMCs

**Fig. 2:**
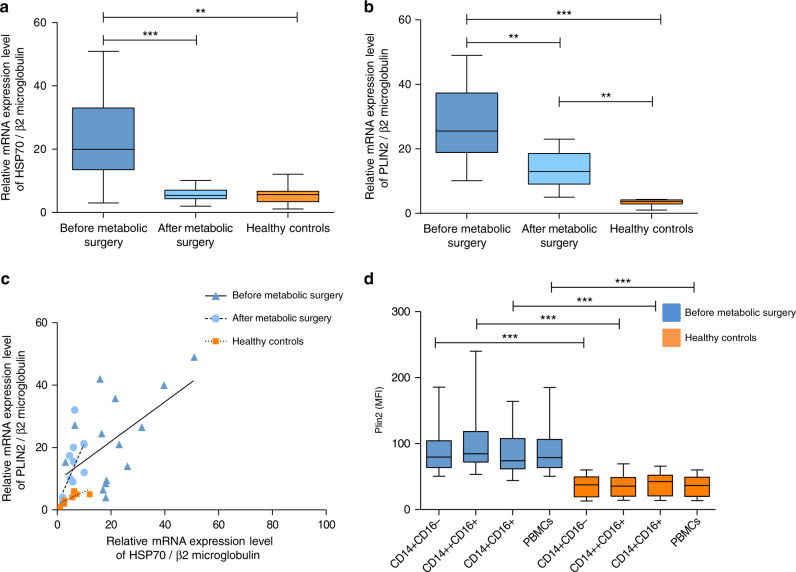
HSP70 and Plin2 mRNA expression in PBMCs are reduced after metabolic surgery. *** P<0.0001, **P<0.007. Data are mean ± s.e.m. or are expressed as median plus minimum and maximum values for whisker plots. **a** HSP70 mRNA expression in PBMCs from obese IR, NAFLD subjects (*n* = 14) before and after metabolic surgery, and in PBMCs from healthy controls (*n* = 12). **b** Plin2 mRNA expression in PBMCs from obese IR, NAFLD subjects (*n* = 14) before and after surgery, and in PBMCs from healthy controls (*n* = 12). **c** Correlation between HSP70 and Plin2 mRNA expressions in PBMCs from obese IR, NAFLD subjects before (*R* = 0.30, *P* = 0.044) and after surgery (*R* = 0.41, *P* = 0.014), and from healthy controls (*R* = 0.56, *P* = 0.0052). **d** Plin2 protein expression (median fluorescence intensity (MFI)) in monocytes and PBMCs from obese, IR subjects (*n* = 14) compared to healthy controls (*n* = 12) (CD14+CD16−: 86.77 ± 7.05 vs. 35.68 ± 4.57, *P* = 0.0001; CD14++CD16+: 98.72 ± 9.15 vs. 36.65 ± 4.59, *P* = 0.0001; CD14+CD16+: 86.52 ± 7.36 vs. 38.58 ± 4.79, *P* = 0.0006; total PBMCs: 86.60 ± 6.97 vs. 35.65 ± 4.41, *P* = 0.0001)

HSP70 and Plin2 messenger RNA (mRNA) expression in PBMCs was quantified by real-time PCR experiments. HSP70 and Plin2 mRNA expressions in PBMCs were strongly reduced after metabolic surgery. HSP70 mRNA expression decreased from 22.80 ± 4.55 to 5.90 ± 0.76 (*P* = 0.002), becoming similar to healthy controls (5.50 ± 1.20, *P* = NS) (Fig. 2a). Plin2 was also significantly reduced after metabolic surgery (from 27.15 ± 3.80 to 13.70 ± 1.81, *P* = 0.006), although it remained higher than in healthy controls (13.70 ± 1.81 vs. 3.25 ± 0.33, *P* = 0.001) (Fig. 2b).

As shown in Fig. 2c, HSP70 and Plin2 mRNA expressions in PBMCs were strongly correlated (before metabolic surgery: *R* = 0.30, *P* = 0.044; after metabolic surgery: *R* = 0.41, *P* = 0.014; healthy controls: *R* = 0.56, *P* = 0.0052).

### Plin2 expression is increased in PBMC and monocytes of IR subjects

To determine if Plin2 protein expression was increased in obese IR, NAFLD subjects, flow cytometry analysis was performed in monocyte subpopulations (CD14+CD16−, CD14++CD16+ and CD14+CD16+) and in total PBMCs of obese IR, NAFLD subjects and of healthy controls. Plin2 protein expression was markedly increased in monocyte subpopulations and PBMCs of obese IR subjects compared to healthy controls (Fig. 2d). Since no significant differences were found in Plin2 expression between monocyte subpopulations and total PBMCs, the stimulation experiments were performed on total PBMC population.

Supplementary Figure 3A shows the gating strategy for Plin2 in monocyte subpopulations.

### HSP70 and GRP78 induce fat accumulation in PBMCs and in HepG2

**Fig. 3:**
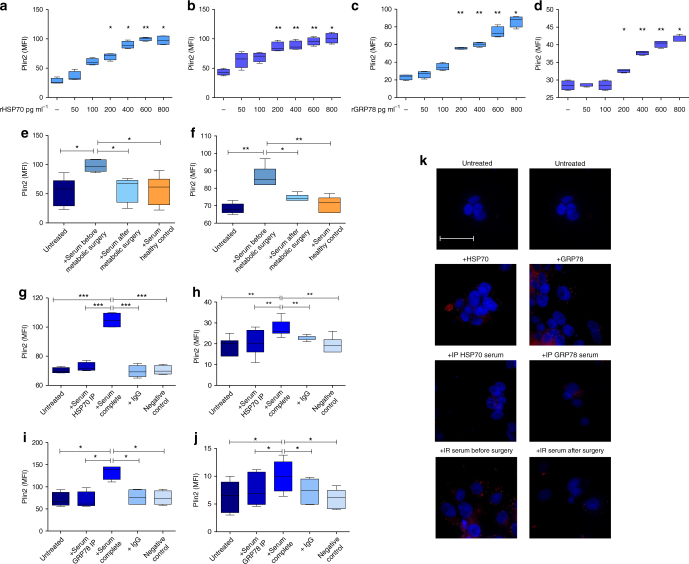
HSP70 and GRP78 induce fat accumulation in PBMCs and HepG2. ^*^P < 0.05, ^**^P < 0.004, ^***^P < 0.0009. Number of samples = 5. Data are mean ± s.e.m. or are expressed as median plus minimum and maximum values for whisker plots. **a**–**d** PBMCs from 5 different healthy controls (**a**, **c**) or 5 batches of HepG2 (**b**, **d**) were incubated with 50–800 pg mL^−1^ of each HSP and the expression of Plin2 measured by flow cytometry. (**a**, **b**) Refer to HSP70, while (**c**, **d**) refer to GRP78. The data are reported in Supplementary Table 1. **e**, **f** Plin2 protein expression (MFI) in PBMCs from healthy controls (**e**) and in HepG2 cells (**f**) is significantly increased after stimulation with serum from obese IR, NAFLD subjects before metabolic surgery. Sera from subjects after metabolic surgery determined effects similar to those of healthy controls (PBMCs: after surgery 57.82 ± 8.48, healthy controls 56.50 ± 10.09, *P* = NS; HepG2: after surgery 74.40 ± 0.93, healthy controls 70.60 ± 2.09, *P* = NS). **g**, **h**, **i**,**j** To exclude that Plin2 increase can be an unspecific effect, PBMCs and HepG2 were stimulated with IgG and with a combination of immunoprecipitation buffer and HSP70/GRP78 antibody (negative control). No effect on Plin2 expression was observed after stimulation with IgG or negative control. PBMCs: serum depleted (immunoprecipitation, IP) of HSP70 72.63 ± 1.52 vs. complete serum 104.8 ± 2.62, *P* = 0.0009 (**g**); HepG2: serum depleted of HSP70 20.21 ± 2.24 vs. complete serum 27.29 ± 1.49, *P* = 0.00235 (**h**); PBMCs: serum depleted of GRP78 69.00 ± 9.74 vs. complete serum 133.5 ± 8.02, *P* = 0.012 (**i**); HepG2: serum depleted of GRP78 7.53 ± 1.17 vs. complete serum 10.01 ± 1.12, *P* = 0.0014 (**j**). **k** Representative images of Nile Red staining in HepG2 untreated, treated with HSP70, GRP78, serum from an obese IR, NAFLD subject before and after metabolic surgery and after immune depletion of HSP70 or GRP78. Scale bar is 50 µm

In order to study the effect of HSPs on intracellular fat accumulation, PBMCs from 5 different healthy controls or 5 batches of HepG2 were incubated with 50–800 pg mL^−1^ of each HSP and the expression of Plin2 measured by flow cytometry (Fig. 3a–d). Supplementary Table 1 reports the quantitative effect of HSP70 and GRP78 on Plin2 expression.

Plin2 median fluorescence intensity (MFI) in PBMCs (Fig. 3e) and in HepG2 (Fig. 3f) significantly increased after stimulation with serum of obese IR, NAFLD subjects before metabolic surgery (PBMCs: 54.1 ± 9.55 vs. 97.67 ± 4.0, *P* = 0.010; HepG2 68.40 ± 1.33 vs. 86.20 ± 2.78, *P* = 0.003). Sera from subjects after metabolic surgery determined effects similar to those of healthy controls.

To confirm that the increased Plin2 expression was entirely related to HSPs, PBMCs and HepG2 were stimulated with serum depleted of HSPs. Stimulation of PBMCs (Fig. 3g–i) and HepG2 (Fig. 3h–**j**) with serum depleted of HSP70 or GRP78 did not change the expression of Plin2, while stimulation with complete serum from the same obese IR, NAFLD subjects increased significantly Plin2 MFI. PBMCs and HepG2 stimulation with IgG and with a combination of immunoprecipitation buffer and HSP70/GRP78 antibody (negative control) was used to exclude that Plin2 increase can be an unspecific effect. No effect on Plin2 expression was observed after stimulation with IgG or negative control.

Supplementary Figure 3B-C shows western blot of supernatants and immune-precipitates from obese serum against HSP70/GRP78 to prove HSP depletion.

Supplementary Figure 3D-G shows Plin2 and lipid droplet gating strategy in PBMCs and HepG2, respectively. Supplementary Figure 4A-B shows propidium iodide staining in PBMCs and HepG2 cells, respectively.

To further investigate lipid accumulation, cells were stained with Nile Red to quantify lipid droplets within HepG2 cells after stimulation with HSP70, GRP78 and serum from obese IR, NAFLD subjects before and after metabolic surgery, and after HSP70/GRP78 immunodepletion (Fig. 3**k**).

The number of lipid droplets in PBMCs and HepG2, quantified by flow cytometry and expressed as MFI, significantly increased after HSP70 and GRP78 stimulation in PBMCs and HepG2 (Supplementary Fig. 5A-D). Supplementary Table 2 reports the quantitative effect of HSP70 and GRP78 on lipid droplets.

Moreover, lipid droplets in PBMCs and HepG2 significantly increased after stimulation with serum of obese IR, NAFLD subjects before metabolic surgery (PBMCs: 2.65 ± 0.15 vs. 4.50 ± 0.147, *P* = 0.023; HepG2 22.9 ± 0.198 vs. 31.89 ± 1.81, *P* = 0.029). Nile Red intensity was significantly reduced after metabolic surgery (PBMCs: before 4.50 ± 0.147 vs. after surgery 2.92 ± 0.063, *P* = 0.008; healthy controls 2.50 ± 0.041; HepG2: before 31.89 ± 1.81 vs. after surgery 26.08 ± 0.584, *P* = 0.040; healthy controls 24.20 ± 0.788) (Supplementary Figure 5E, F).

Finally, PBMC and HepG2 stimulation with serum depleted of HSPs did not increase the lipid droplet number, while stimulation with complete serum from the same obese patients increased significantly intracellular fat accumulation and no effect was observed after stimulation with IgG or negative control (Supplementary Fig. 5G-I-L).

### HSPs inhibit Akt phosphorylation and glucose uptake in HepG2

**Fig. 4:**
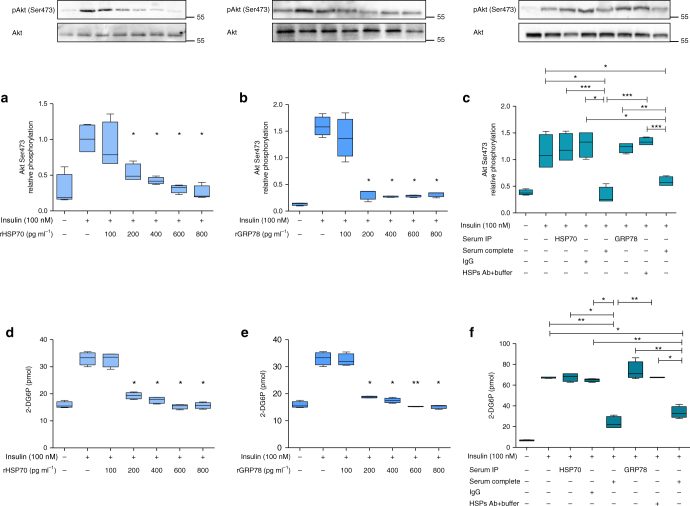
HSP70 and GRP78 inhibit insulin-stimulated Akt phosphorylation and glucose uptake in HepG2. ^*^P < 0.05, ^**^P < 0.009, ^***^P < 0.0009. Number of samples = 5. Data are mean ± s.e.m. or are expressed as median plus minimum and maximum values for whisker plots. Original blot images are reported in the Supplementary Figure 6. **a**, **b** Akt Ser473 relative phosphorylation in HepG2 cells during insulin stimulation (100 nM) is inhibited by HSP70 (**a**) and GRP78 (**b**) in the concentration range of 200–800 pg mL^−1^. **c** Akt Ser473 relative phosphorylation in the insulin-stimulated (100 nM) HepG2 cells is inhibited by complete serum of obese IR, NAFLD subjects before metabolic surgery. Immunodepleted (immunoprecipitation (IP)) serum of HSP70 or GRP78 from the same obese IR, NAFLD subjects did not inhibit Ser473 Akt phosphorylation. **d**, **e** HSP70 (**d**) and GRP78 (**e**) inhibit insulin-mediated (100 nM) 2-DG6P uptake in HepG2 cells. **f** Insulin-mediated (100 nM) 2-DG6P uptake in HepG2 cells is inhibited by complete serum from obese IR, NAFLD subjects before metabolic surgery. Immunedepleted serum of HSP70 or GRP78 from the same obese IR, NAFLD subject did not affect insulin-mediated 2-DG6P uptake

Incubation of HepG2 with HSPs or serum from obese IR, NAFLD subjects inhibited Akt phosphorylation on Ser473 (Fig. 4a–c) and insulin-mediated glucose uptake (Fig. 4d–f), while no effect was detected after stimulation with serum depleted of HSP70 or GRP78. Supplementary Table 3 reports the quantitative effect of HSP70 and GRP78 on Akt phosphorylation and insulin-mediated glucose uptake in HepG2.

## Discussion

Our study shows that: (i)  the jejunum secretes HSP70 and GRP78; (ii) circulating levels of HSP70 are normalized after duodenal–jejunal bypass and there is a good direct correlation with insulin resistance metric and indirect correlation with measures of insulin sensitivity; (iii) a high-calorie meal rich in carbohydrates and fats acutely raises circulating levels of HSP70 and  Plin2 expression in PBMCs; (iv) both HSPs and sera from obese, insulin-resistant subjects impair insulin-mediated glucose uptake in vitro; (v) depletion of serum HSPs using monoclonal antibodies or sera from subjects who underwent duodenum–jejunum bypass restore glucose uptake; (vi) HSPs as well as sera from IR but not insulin-sensitive subjects—such as healthy controls and patients who underwent duodenum–jejunum bypass—increase lipid uptake and storage in both hepatocytes and PBMCs; (vii) depletion of serum HSPs using monoclonal antibodies reverses lipid accumulation; and (viii) PBMC Plin2 gene expression is normalized after the bypass of duodenum and jejunum.

Heat shock proteins, among the most conserved proteins during evolution and of human genome, contain at least 12 genes encoding proteins of the HSP70 family^28^. GRP78 is 64% identical to HSP70^28^.

Traditionally, both HSP70 and GRP78 have been regarded as intracellular chaperones playing important roles in protein folding, maturation and delivery as well as contributing to protein degradation. In this study, we show that HSP70 and GRP78 are released from the gut following in vitro glucose stimulation.

Diet-induced atherosclerosis is indeed associated with a raise in circulating levels of HSP70 in rats^29^ and in our series in humans.

It has been recently shown that the consumption of a high-fat/high-carbohydrate diet causes a gut overgrowth of pro-inflammatory Proteobacteria such as *Escherichia coli* and, simultaneously, a decrease in protective bacteria^30^. A number of bacterial products including the chaperone-60 protein GroEL^31^ and lipopolysaccharides^32^ from *E. coli* stimulate Hsp70 release from PBMCs and caco cells, respectively. The endoplasmic reticulum (ER) stress response also increases the intracellular levels of GRP78 that may exceed the capacity of carboxyl-terminal ER-retention signal (KDEL) to anchor this protein to the ER, resulting in its escape to cell surface and secretion^33^. Another mechanism may involve the deletion of GRP78 KDEL due to alternative splicing, that permits GRP78 secretion^34^.

HSP70 and GRP78 can bind the TLRs stimulating lipogenesis and intracellular fat accumulation^35^ and, in hepatocytes, also gluconeogenesis^36^, therefore promoting insulin resistance. In fact, TLR2/4-dependent activation of JNKs promotes phosphorylation of IRS-1 at Ser307 in rodents and Ser312 in humans leading to inhibition of Akt activation^24^ and, consequently, to an impaired insulin-mediated glucose uptake, similar to what we found in our study.

To support this hypothesis, we demonstrated that HSP70 and GRP78 serum depletion with monoclonal antibodies reverses glucose uptake impairment determined by sera from IR subjects in both HepG2 and PBMCs as well as lipid deposition and Plin2 expression. We found a direct correlation between HSP70 and HOMA-IR, which is a surrogate measure of hepatic insulin resistance. An indirect correlation was instead found between circulating levels of HSP70 and the insulin sensitivity values measured by the oral glucose minimal model.

Duodenal–jeujunal bypass might act through two mechanisms, possibly collaborative: one indirect, involving the exclusion of a large tract of the small intestine from food transit, and one directly related to the lipid malabsorption. In fact, similar to what was reported for fasting^37^, bypassing the duodenum, the whole jejunum and the initial tract of the ileum, duodenal–jeujunal bypass drastically reduces the gut mucosa that enters into contact with gut microbiota’s stress mediator products, resulting in a reduced secretion of HSP70 and GRP78 in response to a high-fat/high-carbohydrate diet with a striking improvement of insulin resistance. Moreover, the lipid malabsorption consequent to bilio-pancreatic juices diversion reduces fat absorption and, consequently, reduces the fat stimulus on endocrine cells leading to a reduced secretion of heat shock proteins.

The major limitation of our study is that we did not prove the causative role of extracellular HSP70 and GRP78, secreted by the small intestine, in insulin resistance by using conditional (intestinal specific) HSP70 and/or GRP78 animal knockout.

In conclusion, a high-energy, high-carbohydrate and high-fat diet can trigger hypersecretion of heat shock proteins from the small intestine that can lead to insulin resistance through inhibition of Akt phosphorylation and insulin-mediated GLUT translocation on cell membrane and ectopic intracellular fat accumulation with NAFLD development. Our results point to gut-secreted HSPs as possible mediators of insulin resistance and suggest that their reduction in the circulatory stream might account for the beneficial effects of metabolic surgery.

## Methods

### Human study design

We enrolled 14 obese IR, NAFLD subjects (according to liver ultrasonography) of both sexes before and 6 months after metabolic surgery. Twelve healthy volunteers served as insulin-sensitive controls. The anthropometric and metabolic variables of obese subjects, before and after metabolic surgery, and healthy controls are reported in Table 1.

Blood samples and PBMCs were taken from all subjects after 12 h of fasting. All obese IR, NAFLD subjects underwent an OGTT before and after metabolic surgery and so did all healthy controls. Five obese IR, NAFLD subjects before metabolic surgery and five healthy controls agreed to undergo a meal test study that was randomly performed in two different days with a standard or a high-energy (high-carbohydrate (CHO) and high-fat) breakfast. Breakfast composition: standard 257 kcal with 25% proteins, 50% CHO and 25% fats; high-energy, high-CHO, high-fat 542 kcal with 5% proteins, 60% CHO and 35% fats.

The study was reviewed and approved by the Ethics Committee of the Catholic University of Rome in accordance with national guidelines and the provisions of the Helsinki Declaration, as revised in 2000. All patients provided written informed consent to participate in the studies, and additional written informed consent was obtained before metabolic surgery.

### Bilio-pancreatic diversion

Our procedure of bilio-pancreatic diversion consists of about 60% distal (horizontal) gastric resection with stapled closure of the duodenal stump. The residual volume of the stomach is about 300 mL. The small bowel is transected at 2.5 m from the ileocecal valve and its distal end is anastomosed to the remaining stomach. The proximal end of the ileum, comprising the remaining small bowel, is anastomosed back to the bowel approximately 50 cm proximal to the ileocecal valve. After bilio-pancreatic diversion, the entirety of the duodenum and jejunum are bypassed and no longer exposed to nutrient flow. The total absorbing bowel is 250 cm in length; of this the proximal 200 cm are exposed to food but not to bile/pancreatic juice, whereas the final 50 cm (distal to the bowel-to-bowel anastomosis) are the only site where nutrient and bile mix again (“common channel”). Because the major physiological modifications are referred to the duodenum and jejunum bypass we will refer to this operation as duodenal–jejunal bypass.

### Intestinal protein secretion

In the five obese IR, NAFLD patients who underwent the meal test, jejunal biopsies have been collected during BPD in order to measure HSPs in the conditioned medium.

Intestinal mucosa was scraped off from the jejunal specimens taken during the operation and incubated in oxygenated (O_2_:CO_2_, 95:5, v/v) Krebs–Henseleit solution (37 °C, pH 7.4) added with complete protease inhibitor cocktail (Roche, Basilea, CH) for 1 h to isolate proteins secreted into the medium. Conditioned medium was lyophilized and stored at −80 °C waiting to be processed. The presence of HSP70 and GRP78 in the conditioned medium was assessed by western blot.

### Oral glucose minimal model and HOMA-IR

The oral glucose minimal model^38^ was used to compute insulin sensitivity (SI) and glucose-effectiveness (SG) in the OGTT and the meal test. We used the glucose, insulin and C-peptide values at fasting and during the test (30, 60, 90, 120 and 180 min) to make calculations. In the subjects who underwent the meal test, serum HSP70, Plin2 and triglycerides were also measured at fasting, and at 60 and 120 min during the test. The dynamic β-cell glucose sensitivity, Φd, the static sensitivity, Φs, and the total sensitivity, Φ, were computed by the C-peptide minimal model^39^. Model parameters were estimated by minimization of a weighted least squares index using an active-set optimization algorithm of the MATLAB library. The variation coefficients of estimates were always <20%. Hepatic insulin resistance was measured by the homeostasis model assessment (HOMA-IR) as fasting insulin (μIU mL^−1^) × fasting glucose (mmol mL^−1^)/22.5^40^.

### Cell cultures

Human HepG2 hepatic carcinoma cells were obtained from the American Type Culture Collection (ATCC, Manassas, VA, USA). HepG2 cells were cultured following the manufacturer’s instructions. Briefly, cells were grown in Dulbecco’s modified Eagle's medium (D-MEM, Gibco/BRL, Carlsbad, CA, USA) supplemented with 10% fetal calf serum (FBS, Gibco/BRL), 100 µg mL^−1^ penicillin and 10 µg mL^−1^ streptomycin (Gibco/BRL) at 37 °C in 5% CO_2_ and were passaged every 5–7 days. HepG2 cells were seeded at equal densities directly into wells of a standard 6-well plate (Nunc, Roskilde, Denmark). Medium was changed every 3–4 days or as required.

PBMCs were obtained from whole blood samples by standard gradient centrifugation over Ficoll-Hypaque (GE Healthcare Bio-Sciences, Piscataway, NJ). PBMCs were rested overnight at 37 °C in 5% CO_2_ in growth medium RPMI (Gibco/BRL, Carlsbad, CA, USA) supplemented with 1% FBS (Gibco/BRL), 100 µg mL^−1^ penicillin and 10 µg mL^−1^ streptomycin (Gibco/BRL). In order to exclude any effect of contamination and to prove that only HSPs were responsible for Plin2 increase and ectopic fat accumulation, we used polymixin B (0.1 μg mL^−1^) for 30 min prior to HSP stimulation.

### Antibodies and reagents

Antibodies against phospho-Akt (Ser473) and Akt (pan) (C67E7) were obtained from Cell Signaling Technology (Danvers, MA). Recombinant human HSP70 and GRP78 were obtained from MyBiosource (San Diego, CA). Nile Red and 4',6-diamidino-2-phenylindole (DAPI) were obtained from Thermo Fisher Scientific (Vantaa, Finland). Plin2 antibody was obtained from LS-BIO (Seattle, WA), AlexaFluor 488 from Life Technology (Carlsbad, CA) and CD16-PeCy7 and CD14-ECD from Beckman Coulter (Brea CA). HSP70 (3A3) and GRP78 (76-E6) antibodies were obtained from Santa Cruz Biotechnology (Dallas, Texas) and Dynabeads® Protein A was obtained from Invitrogen (Waltham, MA, USA).

### Quantitative real-time PCR analysis

The total RNA from frozen PBMCs was extracted using the RNeasy Plus Mini Kit (Qiagen GmbH, Hilden, Germany) according to the indications provided by the company. A small aliquot of total RNA obtained (1 μl) was subjected to qualitative and quantitative control by using the microdrop (Thermo Fisher scientific, Vantaa, Finland). The qualitative and quantitative assessment of the individual samples was determined using dedicated software. The total RNA was reverse transcribed into complementary DNA by using iScript RT (Bio-Rad Laboratories, Hercules, CA). SYBR Green gene expression assays were performed in triplicate according to the manufacturer’s instruction using the iQ™ SYBR® Green Supermix (Bio-Rad Laboratories, Hercules, CA) and the iQ5 Multicolor Real-Time PCR Detection System (Bio-Rad Laboratories, Hercules, CA). For this purpose, the following pairs of primers were used: Plin2 (forward 5’-AGAAGCCAAGTTATTATGTTAGAC-3’ and reverse 5’-TTATCCTGAGCATCCTG-3’) and HSP70 (forward 5’-CCTGAACAAGAGCATCAATCC-3’ and reverse 5’-GAGTAGGTGAAG GTCCAATCCG-3’). The mRNA expression levels were normalized to Beta2 microglobulin (forward 5’-AGGACTGGTCTTTCTATCTCTTGT-3’ and reverse 5’-ACCTCCATGATGCTGCTTACA-3’) and quantification of relative gene expression, presented as percentage of the relevant baseline, was calculated using the 2-∆CT (comparative threshold) method.

### HSP70 ELISA

The blood was collected from obese IR, NAFLD subjects before and 6 months after metabolic surgery and from healthy subjects, and centrifuged at 1000 × *g* for 20 min at 20 °C. The samples were frozen at −80 °C waiting to be processed. HSP70 serum levels were measured with the Human HSP70 enzyme-linked immunosorbent assay (ELISA) Kit (ProteinTech, Chicago, IL) according to the manufacturer’s instructions. Each experiment was performed in duplicate.

### HepG2 stimulation for the assessment of insulin sensitivity

HepG2 cells were cultured overnight at 37 °C in 5% CO_2_ in D-MEM (Gibco/BRL, Carlsbad, CA) without the addition of fetal calf serum. Cells were stimulated with Insulin (100 nmol L^−1^) and recombinant protein (100–800 pg mL^−1^) for 10 min. Stimulation was also performed with HSP70/GRP78 immunoprecipitated serum and complete serum from obese IR, NAFLD subjects.

### Western blot analysis

To analyze the effect of HSP70/GRP78 on Akt Ser473 phosphorylation, HepG2 were rinsed in phosphate buffer saline and homogenized in RIPA buffer containing a cocktail of protease inhibitors. Homogenates were cleared by centrifugation (13.000 rpm; 30 min, 4 °C). Protein content was determined using Bradford Protein Assay (Bio-Rad Laboratories, Hercules,CA). Protein lysates (30 μg) were separated on 8% sodium dodecyl sulfate–polyacrylamide gel electrophoresis and transferred on polyvinylidene difluoride membrane. Membranes were probed overnight with pAkt Ser473 (D9E). Membranes were stripped for 30 min at 56 °C and reprobed overnight with Akt (pan) (C67E7). Detection and analysis were performed respectively with Chemidoc XRS Image system and Image Lab 5.0 software (Bio-Rad Laboratories, Hercules, CA). All the results are expressed as phosphoprotein/total protein ratio. Each experiment was performed in triplicate.

### Glucose uptake assay

To investigate the effect of HSP70/GRP78 on glucose transport, HepG2 were tested for glucose uptake by a commercial kit (Abcam, Cambridge, UK) following the manufacturer’s instructions. Each experiment was performed in duplicate.

### Lipid staining

To assess intracellular lipid accumulation, HepG2 and PBMCs were treated for 24 h with or without rHSPs, with HSP70/GRP78 immunoprecipitated serum and complete serum from obese patients before and after metabolic surgery. Cells were stained for 45 min at 37 °C in 5% CO_2_ with Nile Red (100 ng mL^−1^) and fixed with 4% formalin. Nuclear staining was performed using DAPI (1 µg mL^−1^). Photographs were taken using confocal microscope Spinning Disk; Crest X-Light Confocal Imager (Germany) and MetaMorph Microscopy Automation & Image Analysis Software (Molecular Devices) were used to analyze images.

### Flow cytometry

To assess Plin2 basal expression, PBMCs from obese IR, NAFLD subjects and healthy controls were fixed and permeabilized in FIX/PERM buffer (eBioscience San Diego, CA), and stained for Plin2 using AlexaFluor 488 as secondary antibody. Results were expressed as MFI. To identify monocyte subpopulation, CD16-PeCy7 and CD14-ECD were used. PBMCs and HepG2 stimulated with HSPs (50–800 pg mL^−1^), were fixed and permeabilized in FIX/PERM buffer (eBioscience San Diego, CA) and stained for Plin2 using AlexaFluor 488 as secondary antibody. Flow cytometric analysis was conducted with FC 500 (Beckman Coulter, Brea, CA) and the data analyzed with Kaluza software (Beckman Coulter, Brea, CA).

### HSPs serum immunodepletion

HSP70 and GRP78 immunoprecipitation from obese IR, NAFLD subjects serum was performed using 1.5 mg of Dynabeads® Protein G and 5 μg of HSP70/GRP78 antibody for 1 h at room temperature. Western blot of supernatants and immune-precipitates from obese serum against HSP70/GRP78 were used to prove the depletion of HSP70/GRP78 from serum. Serum immunodepletion of HSP70/GRP78 was performed in five obese IR, NAFLD subjects. Each experiment was performed in triplicate.

### Statistical analysis

Data were expressed as means ± s.e.m. unless specified otherwise. Statistical analyses (SPSS version 13) were performed using Wilcoxon signed ranks test and repeated measures test with Bonferroni’s correction where appropriate. Linear regression analyses were performed for each group of subjects. Two-way repeated measures analysis of variance was used to test differences in circulating levels of serum HSP70 and glucose after the meals. Differences were considered statistically significant at *P* < 0.05. Nonlinear regressions were performed by a MATLAB routine (nlinfit).

### Data availability

The authors declare that all the data supporting the findings of this study are available in the manuscript, figures and supplementary information files.

## Electronic supplementary material

Supplementary Information
