# Supplementary material for: Metabolic surgery improves insulin resistance through the reduction of gut-secreted heat shock proteins
Source: Commun Biol. 2018 Jun 13;1:69. doi: 10.1038/s42003-018-0069-8 (PMC6123703; doi:10.1038/s42003-018-0069-8)
Supplement: Supplementary file 1 — Supplementary Information [file 42003_2018_69_MOESM1_ESM.pdf]

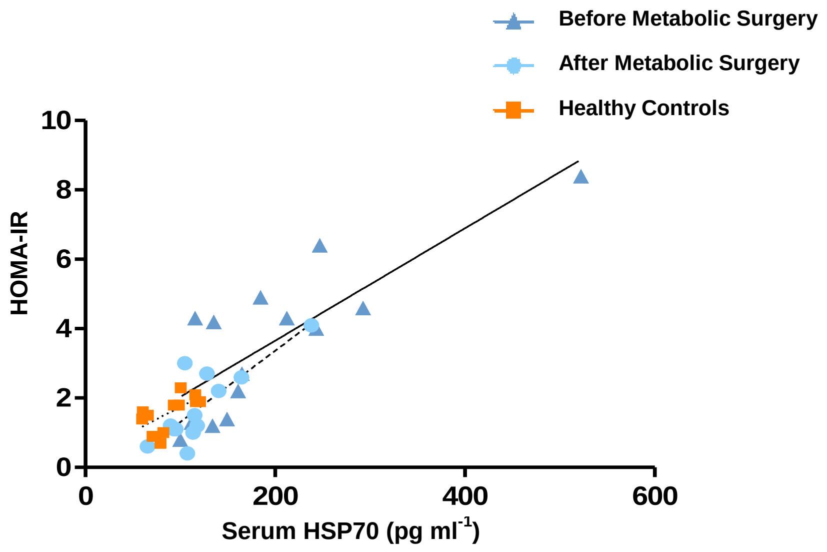


**Supplementary Figure 1. HSP70 circulating levels significantly correlate with HOMA-IR**

Fasting HSP70 circulating levels significantly and positively correlated with HOMA-IR in obese IR subjects (n=14) and healthy controls (n=12) (before metabolic surgery: R=0.39, P=0.029; after metabolic surgery: R=0.66, P=0.0004; healthy controls: R=0.66, P=0.0004).

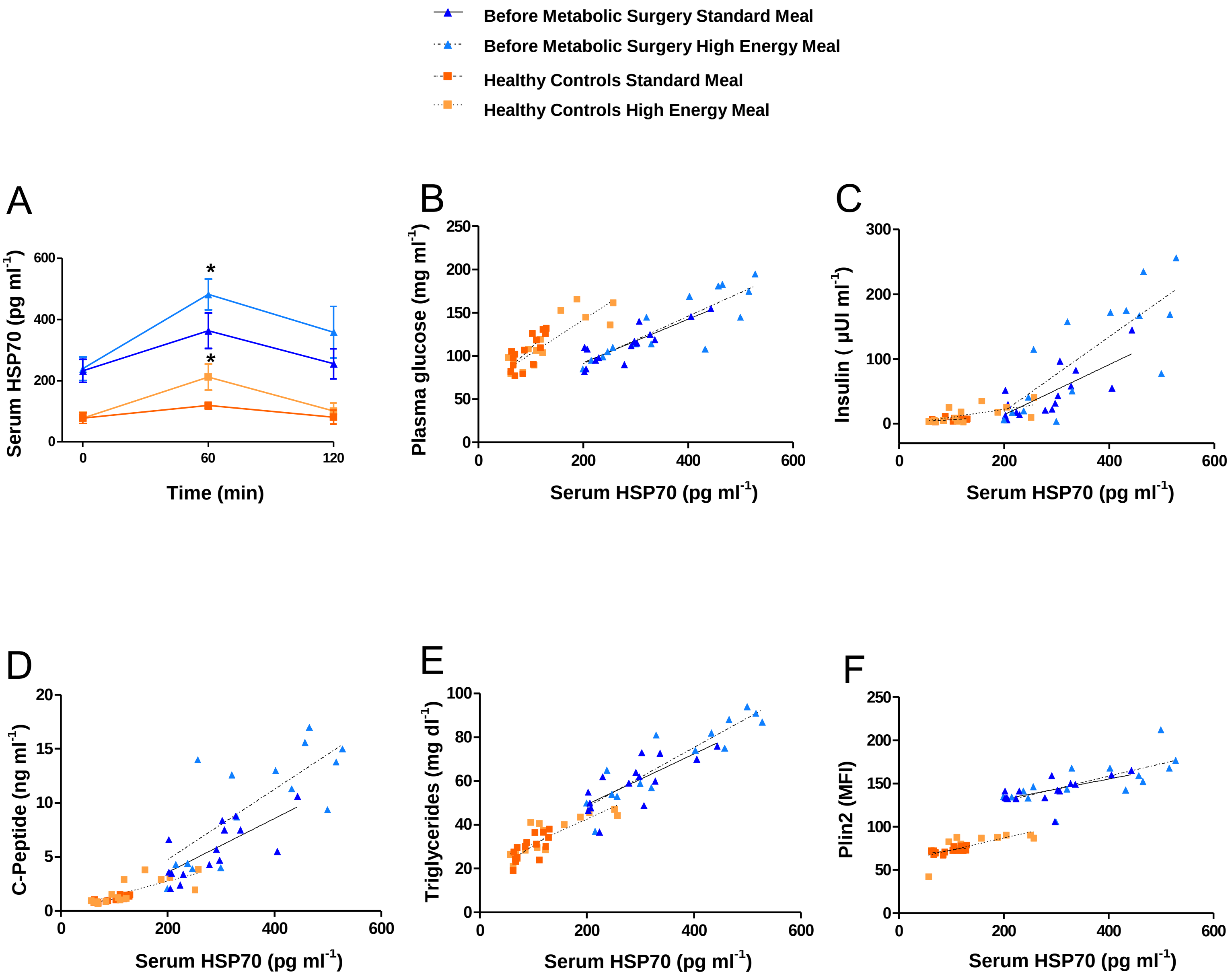


**Supplementary Figure 2. HSP70 circulating levels increase after a high-energy meal**

**Panel A:** High-energy breakfast significantly raised HSP70 serum level after 60 min compared to standard diet (subjects before metabolic surgery, n=5, P=0.027; healthy controls, n=5, P=0.019). **Panels B, C, D, E, F:** HSP70 serum levels, measured after standard or high-energy breakfast at 0, 60 and 120 min in 5 obese IR, NAFLD subjects before surgery and in 5 healthy controls, are plotted vs. plasma glucose, insulin, C-peptide, triglycerides, and Plin2 protein expression in PBMCs. According to linear regression analysis, HSP70 significantly and positively correlated with glucose (subjects before metabolic surgery: standard R=0.74, P<0.0001; high-energy R=0.74, P<0.0001; healthy controls: standard R=0.58, P=0.001; high energy R=0.074, P<0.0001); insulin (subjects before metabolic surgery: standard R=0.60, P=0.0009; high-energy R=0.62, P=0.0005; healthy controls: standard R=0.27, P=0.046; high-energy R=0.42, P=0.009); C-peptide (subjects

before metabolic surgery: standard R=0.54, P=0.0017; high-energy R=0.57, P=0.0012; healthy controls: standard R=0.76, P<0.0001; high-energy R=0.58, P=0.0009); triglycerides (subjects before metabolic surgery: standard R=0.43, P=0.008; high-energy R=0.52, P=0.002; healthy controls: standard R=0.35, P=0.019; high-energy R=0.47, P=0.004); Plin2 (subjects before metabolic surgery: standard R=0.57, P=0.0011 high-energy R=0.82, P<0.0001; healthy controls: standard R=0.52, P=0.0024; high-energy R=0.71, P<0.0001).

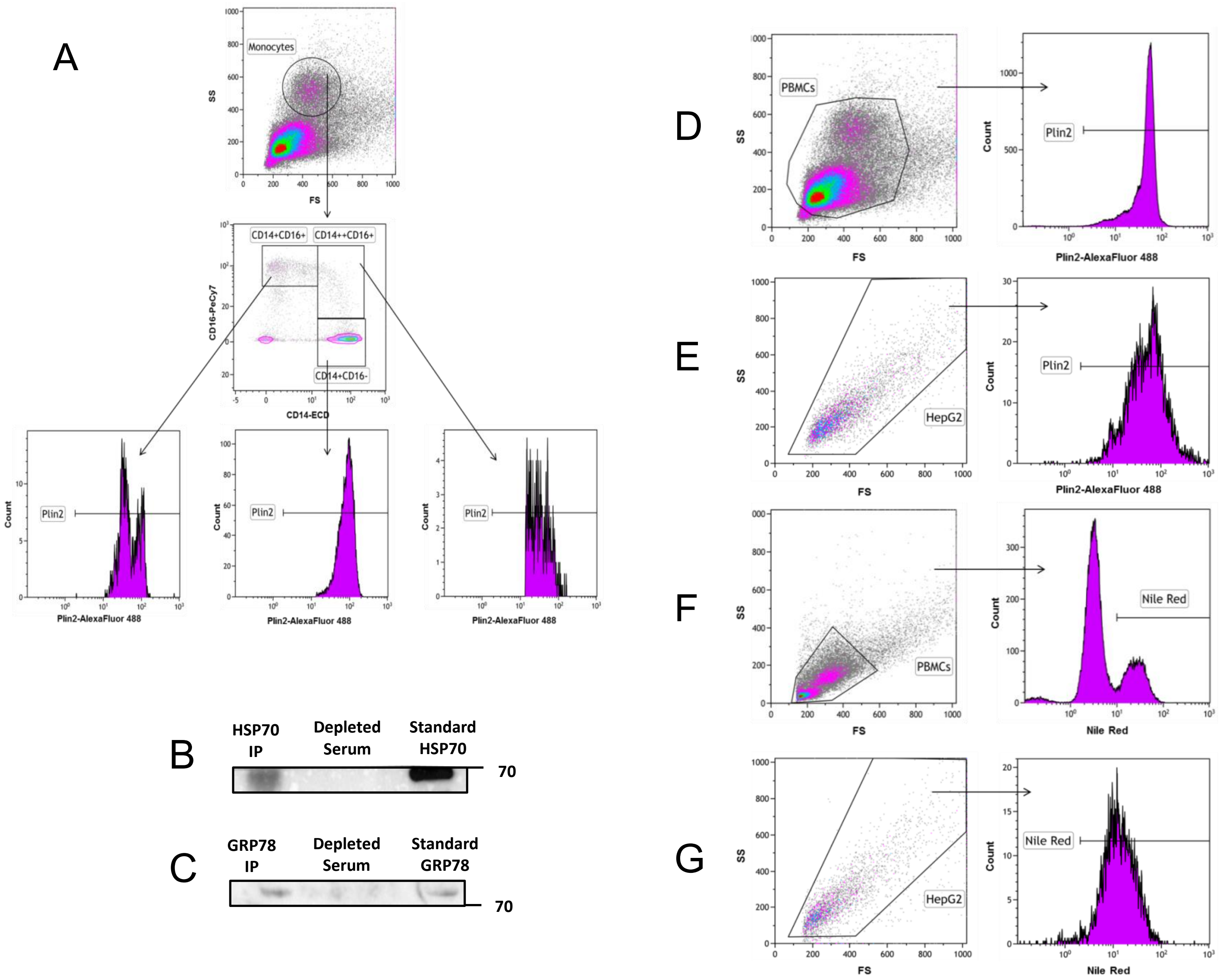


**Supplementary Figure 3. Gating strategy and HSPs immune-depletion**

**Panel A:** Gating strategy for Plin2 in monocyte subpopulations. **Panels B, C:** Representative images of serum depleted of HSP70 (B) and GRP78 (C). Number of experiments n=5. Original blot images presented in Supplementary Figure 6. **Panels D, E*:*** Gating Strategy for Plin2 quantification by flow cytometry in PBMCs (D) and HepG2 (E). **Panels F, G*:*** Gating Strategy for lipid droplets quantification by flow cytometry in PBMCs (F) and HepG2 (G).

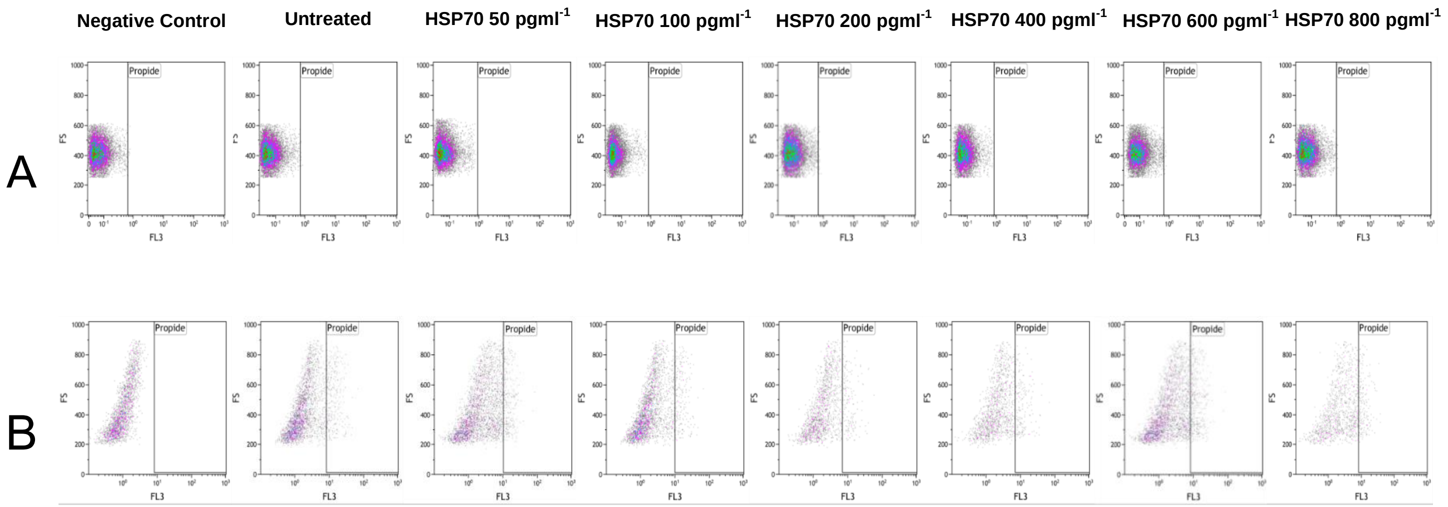


**Supplementary Figure 4. Viability staining**

***Panels A, B:*** Representative image of propidium iodide staining in PBMCs (A) and HepG2 (B) after 24-h stimulation with HSPs.

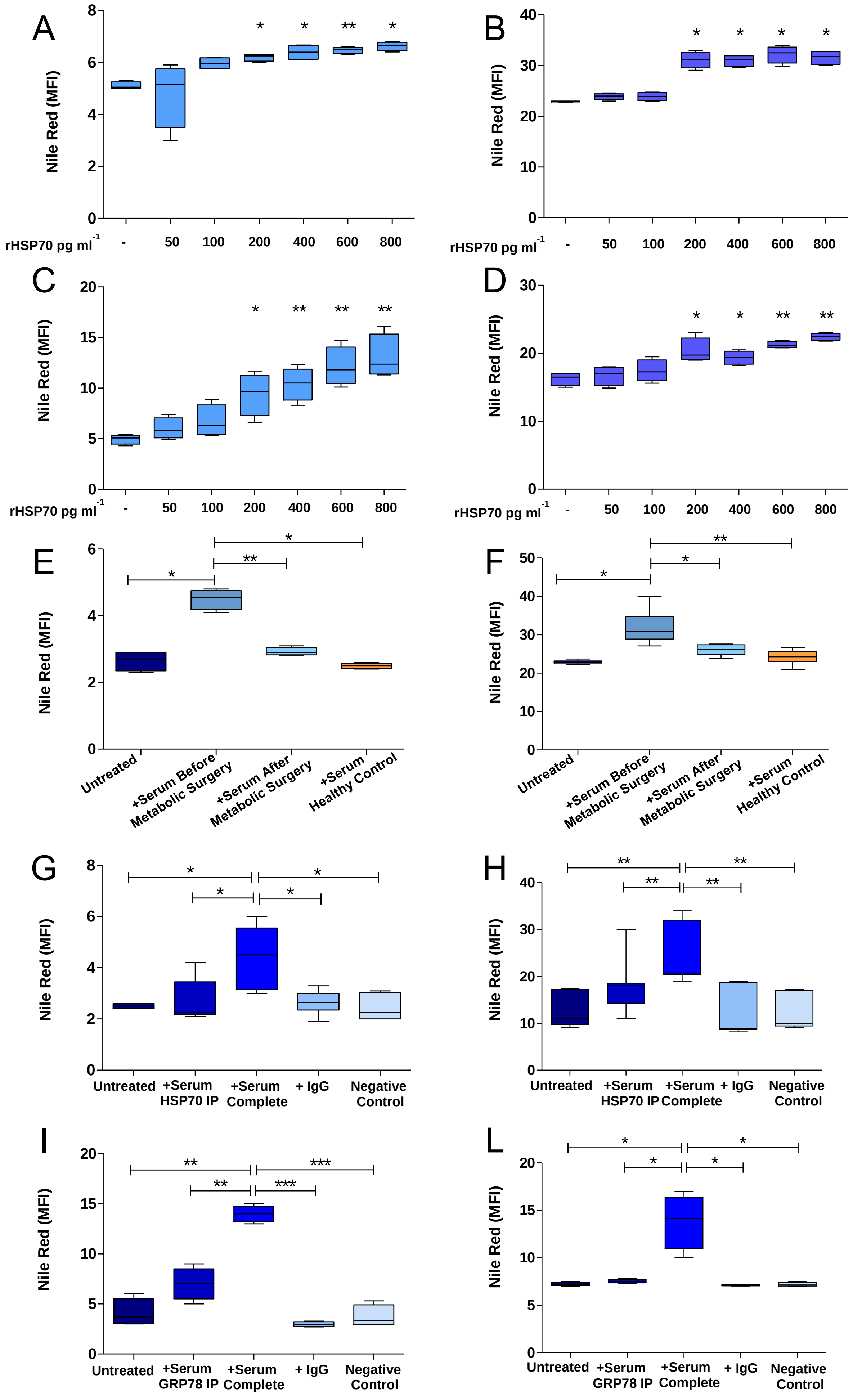

A
B
C
D
E
F
G
H
I
L
Nile Red (MFI)
rHSP70 pg ml-1
- 50 100 200 400 600 800
Untreated
+Serum Before Metabolic Surgery
+Serum After Metabolic Surgery
+Serum Healthy Control
+Serum HSP70 IP
+Serum Complete
+ IgG
Negative Control
+Serum GRP78 IP
*
**
***

**Supplementary Figure 5. HSP70 and GRP78 induce fat accumulation in PMBCs and in HepG2**

**Panels A, B, C, D:** The number of lipid droplets (MFI) in PBMCs from healthy controls (A-C) and in HepG2 cells (B-D) is significantly increased after stimulation with HSP70 or GRP78. **Panels E, F*:*** The number of lipid droplets in PBMCs from healthy controls (E) and in HepG2 cells (F) is significantly increased after stimulation with serum from obese IR, NAFLD subjects before surgery, while serum from the same subjects after surgery and from healthy controls did not change Nile Red intensity. **Panels G, H, I, L:** The number of lipid droplets (MFI) increases in PBMCs (G-I) and in HepG2 cells (H-L) stimulated with complete serum from obese IR, NAFLD subjects, while immunedepleted serum of HSP70 (G, H) or GRP78 (I, L) from the same obese IR, NAFLD subjects did not change Nile Red intensity. No effect was observed after stimulation with IgG or negative control. *P*-value legend: $^{*}P<0.05$, $^{**}P<0.009$, $^{***}P<0.0009$. Number of samples = 5.

Data are mean± s.e.m. or are expressed as median plus minimum and maximum values for whisker plots.

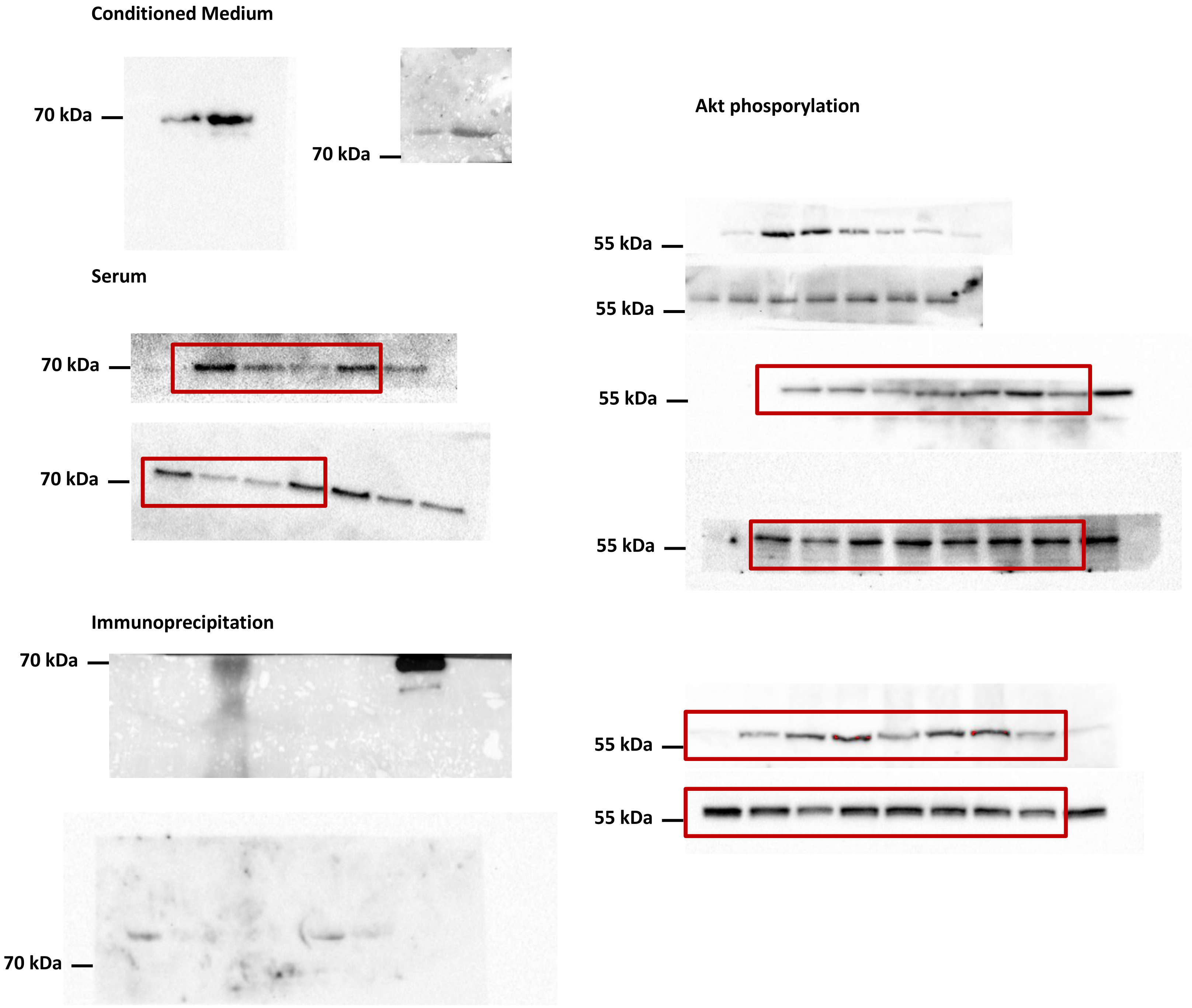


**Supplementary Figure 6**

Full blot images for cropped gels. The red-boxed regions are used in Figure 1 Panel-A and Figure 4 Panels-B-C.

**Supplementary Table 1.** Statistical data (Mean ±s.e.m.) of Plin2 expression (MFI) in PBMCs (top) and HepG2 (bottom).

| Conditions | Mean±s.e.m. | P value |
|---|---|---|
| **Untreated vs HSP70 50pg/ml** | 27.70±2.49 vs 36.07±4.02 | NS |
| **Untreated vs HSP70 100pg/ml** | 27.70±2.49 vs 60.82±2.79 | NS |
| **Untreated vs HSP70 200pg/ml** | 27.70±2.49 vs 70.00±2.79 | 0.027 |
| **Untreated vs HSP70 400pg/ml** | 27.70±2.49 vs 89.47±3.13 | 0.025 |
| **Untreated vs HSP70 600pg/ml** | 27.70±2.49 vs 99.70±1.39 | 0.001 |
| **Untreated vs HSP70 800pg/ml** | 27.70±2.49 vs 97.65±3.97 | 0.023 |
| | | |
| **Untreated vs GRP78 50pg/ml** | 23.30±1.52 vs 26.27±1.90 | NS |
| **Untreated vs GRP78 100pg/ml** | 23.30±1.52 vs 34.67±1.96 | NS |
| **Untreated vs GRP78 200pg/ml** | 23.30±1.52 vs 55.87±0.049 | 0.002 |
| **Untreated vs GRP78 400pg/ml** | 23.30±1.52 vs 60.17±1.19 | 0.009 |
| **Untreated vs GRP78 600pg/ml** | 23.30±1.52 vs 73.97±2.99 | 0.008 |
| **Untreated vs GRP78 800pg/ml** | 23.30±1.52 vs 86.40±3.13 | 0.011 |

| Conditions | Mean± s.e.m. | P value |
|---|---|---|
| **Untreated vs HSP70 50pg/ml** | 43.33±2.66 vs 64.10±6.48 | NS |
| **Untreated vs HSP70 100pg/ml** | 43.33±2.66 vs 69.15±3.90 | NS |
| **Untreated vs HSP70 200pg/ml** | 43.33±2.66 vs 86.00±4.08 | 0.007 |
| **Untreated vs HSP70 400pg/ml** | 43.33±2.66 vs 88.30±3.79 | 0.005 |
| **Untreated vs HSP70 600pg/ml** | 43.33±2.66 vs 95.60±3.28 | 0.008 |
| **Untreated vs HSP70 800pg/ml** | 43.33±2.66 vs 101.0±4.21 | 0.042 |
| | | |
| **Untreated vs GRP78 50pg/ml** | 28.50±0.64 vs 28.75±0.25 | NS |
| **Untreated vs GRP78 100pg/ml** | 28.50±0.64 vs 28.50±0.64 | NS |
| **Untreated vs GRP78 200pg/ml** | 28.50±0.64 vs 32.63±0.24 | 0.018 |
| **Untreated vs GRP78 400pg/ml** | 28.50±0.64 vs 37.68±0.27 | 0.002 |
| **Untreated vs GRP78 600pg/ml** | 28.50±0.64 vs 40.25±0.48 | 0.001 |
| **Untreated vs GRP78 800pg/ml** | 28.50±0.64 vs 41.50±0.50 | 0.010 |

**Supplementary Table 2.** Statistical data (Mean ± s.e.m.) of Nile Red expression (MFI) in PBMCs (top) and HepG2 (bottom).

| Conditions | Mean± s.e.m. | P value |
|---|---|---|
| **Untreated vs HSP70 50pg/ml** | 5.10±0.071 vs 4.80±0.63 | NS |
| **Untreated vs HSP70 100pg/ml** | 5.10±0.071 vs 5.97±0.11 | NS |
| **Untreated vs HSP70 200pg/ml** | 5.10±0.071 vs 6.2±0.071 | 0.026 |
| **Untreated vs HSP70 400pg/ml** | 5.10±0.071 vs 6.39±0.14 | 0.022 |
| **Untreated vs HSP70 600pg/ml** | 5.10±0.071 vs 6.47±0.063 | 0.007 |
| **Untreated vs HSP70 800pg/ml** | 5.10±0.071 vs 6.62±0.085 | 0.042 |
| | | |
| **Untreated vs GRP78 50pg/ml** | 4.97±0.239 vs 6.00±0.521 | NS |
| **Untreated vs GRP78 100pg/ml** | 4.97±0.239 vs 6.700±0.787 | NS |
| **Untreated vs GRP78 200pg/ml** | 4.97±0.239 vs 9.40±1.06 | 0.012 |
| **Untreated vs GRP78 400pg/ml** | 4.97±0.239 vs 10.40±0.820 | 0.003 |
| **Untreated vs GRP78 600pg/ml** | 4.97±0.239 vs 12.10±0.963 | 0.003 |
| **Untreated vs GRP78 800pg/ml** | 4.97±0.239 vs 13.03±1.09 | 0.003 |

| Conditions | Mean± s.e.m. | P value |
|---|---|---|
| **Untreated vs HSP70 50pg/ml** | 22.94±0.040 vs 23.90±0.033 | NS |
| **Untreated vs HSP70 100pg/ml** | 22.94±0.040 vs 23.92±0.394 | NS |
| **Untreated vs HSP70 200pg/ml** | 22.94±0.040 vs 31.09±0.800 | 0.039 |
| **Untreated vs HSP70 400pg/ml** | 22.94±0.040 vs 30.98±0.564 | 0.014 |
| **Untreated vs HSP70 600pg/ml** | 22.94±0.040 vs 32.23±0.854 | 0.037 |
| **Untreated vs HSP70 800pg/ml** | 22.94±0.040 vs 31.61±0.673 | 0.022 |
| | | |
| **Untreated vs GRP78 50pg/ml** | 16.25±0.48 vs 16.73±0.71 | NS |
| **Untreated vs GRP78 100pg/ml** | 16.25±0.48 vs 17.41±0.81 | NS |
| **Untreated vs GRP78 200pg/ml** | 16.25±0.48 vs 20.38±0.89 | 0.018 |
| **Untreated vs GRP78 400pg/ml** | 16.25±0.48 vs 19.36±0.49 | 0.040 |
| **Untreated vs GRP78 600pg/ml** | 16.25±0.48 vs 21.26±0.24 | 0.006 |
| **Untreated vs GRP78 800pg/ml** | 16.25±0.48 vs 22.44±0.26 | 0.001 |

**Supplementary Table 3**. Statistical data (Mean ± s.e.m.) of relative Akt Ser473 phosphorylation (top) and insulin-mediated glucose-uptake (bottom) in HepG2.

| Conditions | Mean± s.e.m. | P value |
|---|---|---|
| Insulin vs HSP70 100pg/ml | 1.04±0.124 vs 0.98±0.202 | NS |
| Insulin vs HSP70 200pg/ml | 1.04±0.124 vs 0.56±0.076 | 0.027 |
| Insulin vs HSP70 400pg/ml | 1.04±0.124 vs 0.42±0.035 | 0.035 |
| Insulin vs HSP70 600pg/ml | 1.04±0.124 vs 0.31±0.040 | 0.032 |
| Insulin vs HSP70 800pg/ml | 1.04±0.124 vs 0.27±0.066 | 0.020 |
| Insulin vs GRP78 100pg/ml | 1.59±0.128 vs 1.37±0.265 | NS |
| Insulin vs GRP78 200pg/ml | 1.59±0.128 vs 0.30±0.065 | 0.021 |
| Insulin vs GRP78 400pg/ml | 1.59±0.128 vs 0.27±0.008 | 0.010 |
| Insulin vs GRP78 600pg/ml | 1.59±0.128 vs 0.28±0.014 | 0.011 |
| Insulin vs GRP78 800pg/ml | 1.59±0.128 vs 0.31±0.031 | 0.014 |
| Insulin vs HSP70 complete serum | 1.23±0.197 vs 0.35±0.099 | 0.018 |
| Insulin vs GRP78 complete serum | 1.23±0.197 vs 0.61±0.05 | 0.013 |
| HSP70 complete serum vs HSP70 IP serum | 0.35±0.099 vs 1.29±0.160 | 0.0007 |
| GRP78 complete serum vs GRP78 IP serum | 0.61±0.05vs 1.20±0.056 | 0.003 |
| HSP70 complete serum vs IgG | 0.35±0.099 vs 1.21±0.147 | 0.014 |
| HSP70 complete serum vs Negative control | 0.35±0.099 vs 1.36±0.043 | 0.0002 |
| GRP78 complete serum vs IgG | 0.61±0.053 vs 1.21±0.147 | 0.020 |
| GRP78 complete serum vs Negative Control | 0.61±0.053 vs 1.36±0.043 | 0.0001 |

| Conditions | Mean± s.e.m. | P value |
|---|---|---|
| Insulin vs HSP70 100pg/ml | 33.05±1.17 vs 32.73±1.31 | NS |
| Insulin vs HSP70 200pg/ml | 33.05±1.17 vs 19.29±0.591 | 0.042 |
| Insulin vs HSP70 400pg/ml | 33.05±1.17 vs 17.71±0.588 | 0.036 |
| Insulin vs HSP70 600pg/ml | 33.05±1.17 vs 15.32±0.432 | 0.022 |
| Insulin vs HSP70 800pg/ml | 33.05±1.17vs 15.68±0.549 | 0.002 |
| Insulin vs GRP78 100pg/ml | 33.05±1.17 vs 32.43±1.10 | NS |
| Insulin vs GRP78 200pg/ml | 33.05±1.17 vs 18.77±0.177 | 0.024 |
| Insulin vs GRP78 400pg/ml | 33.05±1.17 vs 17.47±0.451 | 0.025 |
| Insulin vs GRP78 600pg/ml | 33.05±1.17 vs 15.25±0.055 | 0.009 |
| Insulin vs GRP78 800pg/ml | 33.05±1.17 vs 15.21±0.332 | 0.011 |
| Insulin vs HSP70 complete serum | 67.23±0.335 vs 26.29±2.70 | 0.008 |
| Insulin vs GRP78 complete serum | 67.23±0.335 vs 34.00±3.99 | 0.012 |
| HSP70 complete serum vs HSP70 IP serum | 26.29±2.70 vs 67.19 ±1.81 | 0.019 |
| GRP78 complete serum vs GRP78 IP serum | 34.00±3.99 vs 74.57±6.08 | 0.003 |
| HSP70 complete serum vs IgG | 26.29±2.70 vs 65.60±1.14 | 0.015 |
| HSP70 complete serum vs Negative control | 26.29±2.70 vs 67.63±0.246 | 0.007 |
| GRP78 complete serum vs IgG | 34.00±3.99 vs 65.60±1.14 | 0.009 |
| GRP78 complete serum vs Negative Control | 34.00±3.99 vs 67.63±0.246 | 0.014 |
